# Prescription patterns of supportive care medications among children receiving chemotherapy treatments at a major referral hospital in Tanzania: where are we in managing chemotherapy-induced toxicities?

**DOI:** 10.3389/fonc.2025.1444565

**Published:** 2025-02-25

**Authors:** Deogratias M. Katabalo, Melina Abraham, Benson R. Kidenya, Antony Liwa, Kristin Schroeder

**Affiliations:** ^1^ Department of Pharmaceutics and Pharmacy Practice, School of Pharmacy, Catholic University of Health and Allied Sciences, Mwanza, Tanzania; ^2^ Department of Oncology, Bugando Medical Centre, Mwanza, Tanzania; ^3^ Department of Biochemistry, School of Medicine, Catholic University of Health and Allied Sciences, Mwanza, Tanzania; ^4^ Department of Pharmacology, School of Medicine, Catholic University of Health and Allied Sciences, Mwanza, Tanzania; ^5^ Duke Global Health Institute, Duke University, Durham, NC, United States

**Keywords:** prescription patterns, pediatrics, supportive care medications, chemotherapy-induced toxicities, Tanzania

## Abstract

**Background:**

Cancer chemotherapy is a treatment that systematically kills cancer cells but causes expected side effects, known as chemotherapy-induced toxicities. These toxicities are managed with supportive care medications. This study aimed to determine the prescription patterns of supportive care medications in children receiving chemotherapy at a major referral hospital in Tanzania.

**Methodology:**

A hospital-based descriptive cross-sectional study was conducted at Bugando Medical Centre (BMC). The study analyzed 104 prescription slips of pediatric cancer patients receiving chemotherapy and qualitatively assessed national guidelines and disease-specific protocols used in guiding treatment. Data were cleaned in Microsoft Excel, analyzed using STATA version 15, and presented as frequencies, percentages, and narrative summaries.

**Results:**

Ondansetron (84.6%) and pre-hydration normal saline (20.2%) were the most prescribed pre-chemotherapy supportive care medications. Similarly, oral ondansetron (80.8%) and post-hydration normal saline (22.1%) were the most prescribed post-chemotherapy medications. Few prescriptions included a combination of antiemetics, fluids, and proton pump inhibitors for regimens with multiple chemotherapeutic agents. National cancer treatment guidelines lacked detailed sections on supportive care medications, leaving prescribing decisions to clinicians, while Burkitt’s lymphoma and nephroblastoma protocols offered more detailed guidance.

**Conclusion:**

Antiemetics and hydration fluids dominated supportive care prescriptions. Significant gaps were identified in the inclusion of supportive care in national guidelines, with reliance on disease-specific protocols. These findings highlight the need for standardized, evidence-based supportive care guidelines tailored to resource-limited settings.

## 1 Background

Cancer chemotherapy is a treatment that uses drugs to treat cancer disease by systematically killing fast-growing cells in the body. Their mechanism of action relies on the behaviors of cancer cells, which tend to grow faster than most normal cells in the body. The other distinct feature of cancer cells is that they have lost functions and tend to invade other parts of the body, resulting in the development of swelling (tumors) or liquid cancer in the body fluids. This is in contrast to fast-growing normal cells, which retain their functions and don’t spread (1). Every year, approximately 400,000 children and adolescents 0–19 years of age develop cancer globally. These forms of cancer, also known as childhood cancers, more often affect lymphatic systems (lymphoma), blood (leukemia), brain tissues (brain cancer), and other solid tissues (nephroblastoma, neuroblastoma, retinoblastoma, rhabdomyosarcoma, osteosarcoma, and others (2). With the growing population, changes in lifestyles, and advancements in diagnostic procedures, 50% of the global burden of childhood cancer is expected to be found in sub-Saharan Africa by 2050 (3). In Tanzania, estimates show that the incidence of childhood cancer is 134 per million (4).

Chemotherapies are the cornerstone of childhood cancer treatment, often used alone or in combination with other modalities such as surgery, radiation, and/or biologics, depending on the type of cancer being treated (5). However, their administration is associated with various adverse effects, collectively known as chemotherapy-induced toxicities (CITs). These toxicities necessitate the use of supportive care medications to prevent or mitigate their impact and enhance treatment outcomes (6). Common toxicities include bone marrow suppression, which leads to immune decline and manifests as febrile neutropenia, anemia, and fatigue. Additionally, nausea and vomiting, which occur with varying severity across all chemotherapies, can cause significant distress and deter treatment adherence. Certain agents, such as anthracyclines are associated with specific toxicities like cardiotoxicity which can result in severe cardiac dysfunction and high morbidity in pediatric patients (7–9). Supportive care measures are critical to addressing these challenges and ensuring effective cancer management.

The prescribed supportive care medications are indicated for prevention (prophylactic) and treatment of these toxicities. However, their efficacy is influenced by many factors, such as the type of medication in relation to the chemotherapy given, the dose, the route of administration, timing during chemotherapy, the type and stage of cancer, potential interactions, and the general condition of the patient (10). Therefore, this study aimed to evaluate the prescription pattern of supportive care medications in relation to chemotherapy given to children undergoing treatment at Bugando Medical Centre (BMC).

## 2 Methods

### 2.1 Study area

The study was conducted at the oncology department of the Bugando Medical Centre, the largest referral and university teaching hospital in the Lake Zone region, in the north-western part of Tanzania. The hospital offers services to over 8 million patients referred from other hospitals in a catchment area occupied by over 16 million people. Being the only hospital with cancer services in the region, it was an ideal area for data collection because all patients and their information are found at the cancer center.

### 2.2 Study design

A descriptive cross-sectional design was used, whereby the prescription information on the slips of all children undergoing outpatient cancer treatment at BMC as well as information from all treatment guidelines and protocols were assessed, collected, and analyzed to generate answers to the objectives of this study.

### 2.3 Study population

Prescription slips of all children undergoing cancer treatment at the BMC oncology department. Also, the national cancer treatment guidelines and disease-specific treatment protocols used at the center were assessed to understand their suggestions, which were then related to the current practices.

### 2.4 Selection criteria

#### 2.4.1 Inclusion criteria

This study included prescription slips for all children under 18 years old undergoing cancer treatment at BMC. Additionally, treatment guidelines and disease-specific protocols, along with their publication sources and dates, were incorporated.

#### 2.4.2 Exclusion criteria

Prescription slips of children aged less than 18 years old undergoing cancer treatment, missing some important information such as age, type, dose, and indication, were excluded from this study. Also, treatment guidelines and protocols missing the publisher’s information or protocols used for research purposes were excluded.

### 2.5 Sample size and sampling procedure

The minimum sample size of the study was calculated using the Taro Yamane formula (1967). The population size was 148 based on the registered active outpatient children undergoing treatment per year. After calculations, a total of 108 prescription slips were set as a sample used to extract data for this study. Then, convenience sampling was used, by including all prescriptions that were issued during the study period, and a sample size was reached.

### 2.6 Study period and rationale for sampling

This study was conducted over three months, from April to June 2024, and analyzed prescription slips issued during the one-year period, from January to December 2023. Approximately 155-250 prescriptions were written during this time for pediatric cancer patients actively undergoing treatment. This is when considering one prescription per patient, but often these patients visit the hospital several times and receive prescriptions. Not all prescriptions were included in the analysis due to logistical issues. A calculated minimum sample size of 108, determined using the Taro Yamane formula (1967), was sufficient to achieve the study objectives while maintaining methodological rigor.

### 2.7 Data collection procedure

Data was collected using a standard checklist carrying items, which was used to gather necessary information from the prescription slips of the patients. This information included demographic information, disease types and stages, chemotherapy given, and support medications prescribed. The other checklist was used to collect information from guidelines and protocols, including the name of the guideline/protocol, year of publication, source, and descriptions of supportive care medications.

### 2.8 Definition and scope of supportive care

In this study, supportive care was defined according to the Multinational Association of Supportive Care in Cancer (MASCC) as interventions aimed at preventing, mitigating, or managing the adverse effects of chemotherapy to improve patient comfort, safety, and treatment outcomes. Supportive care encompasses a broad range of measures, including antiemetics, Colony-stimulating factors (CSFs), analgesics, hydration, and nutritional support (11). Hydration was classified as supportive care in this context due to its primary role in preventing or mitigating chemotherapy-induced nephrotoxicity. This was particularly relevant for protocols involving nephrotoxic agents such as cisplatin, carboplatin, cyclophosphamide, ifosfamide, mitomycin C, high-dose etoposide, and high-dose methotrexate. While not all chemotherapy protocols require pre- and post-hydration, this study assessed hydration in all and in participants undergoing regimens for which it is recommended. Similarly, antiemetics was assessed in patients on regimens that require antiemetics.

### 2.9 Data analysis procedure and statistical analysis

Data was stored in the Microsoft Excel spreadsheet, where it was initially cleaned and coded, then transferred to STATA version 15. Continuous variables were presented as medians with an interquartile range and categorical variables as frequencies and/or percentages.

### 2.10 Ethical consideration

Ethical clearance for this study was obtained from the joint CUHAS/BMC Ethics and Review Committee (Clearance Certificate No. CREC/793/2024). Additionally, permission to conduct the study was granted by the Director General of BMC. Confidentiality was maintained in accordance with the code of ethics and professional conduct.

## 3 Results

The patient’s information was extracted from a total of 108 prescription slips. However, at the data analysis stage four, prescriptions were discarded as they were found to be duplicates of patients already included.

### 3.1 Social demographic information of participants

A total of 104 pediatric cancer patient prescriptions for chemotherapy and supportive care medications were analyzed. The majority of the prescriptions, 59 (56.7%), were for male patients, while 45 (43.3%) were for female patients. The median age of the patients was 6 years (IQR = 8.5). The largest age group consisted of children aged 3–6 years, accounting for 42 (40.4%) of the patients, followed by 27 (25.9%) aged 7–12 years, 22 (21.2%) aged over 12 years, and 13 (12.5%) aged 0–2 years.

### 3.2 Types and stages/grades/risk of oncological conditions diagnosed in pediatric patients receiving treatment at Bugando Medical Centre

A total of 22 types of oncological conditions were observed in the prescriptions, with nephroblastoma being the most prevalent (20.2%, n = 21), followed by acute lymphoblastic leukemia (ALL) (13.5%, n = 14) and Hodgkin’s lymphoma (11.5%, n = 12), as shown in **Table 1**. Hemangioma, a non-cancerous condition, was also included and accounted for one patient (This patient was on chemotherapy). Among patients with solid tumors, the majority (36.5%, n = 38) were in stage II, while those with nervous system tumors (pilocytic astrocytoma and brain tumor) were primarily classified as low grade (4.8%, n = 5). Additionally, ALL was the predominant type among patients with hematological malignancies (16.4%, n = 17), with the majority (8.7%, n = 9) presenting with high-risk disease, as shown in **Table 2**.

**Table 1:** Types of cancer/oncological conditions diagnosed in children receiving treatment at Bugando Medical Centre.

Diagnosis		Frequency	Percentage (%)
Leukemias	Acute lymphoblastic leukemia	14	13.5
	Acute myeloid leukemia	3	2.9
Lymphomas	Hodgkin’s lymphoma	12	11.5
	Burkitt lymphoma	11	10.6
	Diffuse large B cell lymphoma	3	2.9
	Intra-abdominal small-cell lymphoma	1	1.0
Solid tumors	Nephroblastoma	21	20.2
	Retinoblastoma	11	10.6
	Rhabdomyosarcoma	8	7.7
	Osteosarcoma	4	3.9
	Ewing’s sarcoma	2	1.9
	Malignant peripheral nerve sheath tumor	2	1.9
	Neuroblastoma	2	1.9
	Hepatoblastoma	1	1.0
	Hepatocellular carcinoma	1	1.0
	Left leg pleomorphic liposarcoma	1	1.0
	Nasopharyngeal carcinoma	1	1.0
	Synovial sarcoma	1	1.0
	Hemangioma	1	1.0
Central nervous system tumors	Brain tumor	3	2.9
	Pilocytic astrocytoma	1	1.0

**Table 2:** Stages, grades, and risks of cancer among pediatric patients receiving treatment at Bugando Medical Centre.

Variable	Description of a variable	Frequency	Percentage
Stages of solid tumors presented by participants	Stage I	2	1.9
	Stage II	28	36.5
	Stage III	25	24.0
	Stage IV	16	15.4
Grades of central nervous tumors presented by participants	Low grade	5	4.8
	High grade	1	1.0
Risks of hematological malignancies presented by participants	Low risk	6	5.8
	Standard risk	2	1.9
	High risk	9	8.7

### 3.3 Type, dose, and indications of medications prescribed as pre- and post-chemotherapy supportive care

**Figure 1:**
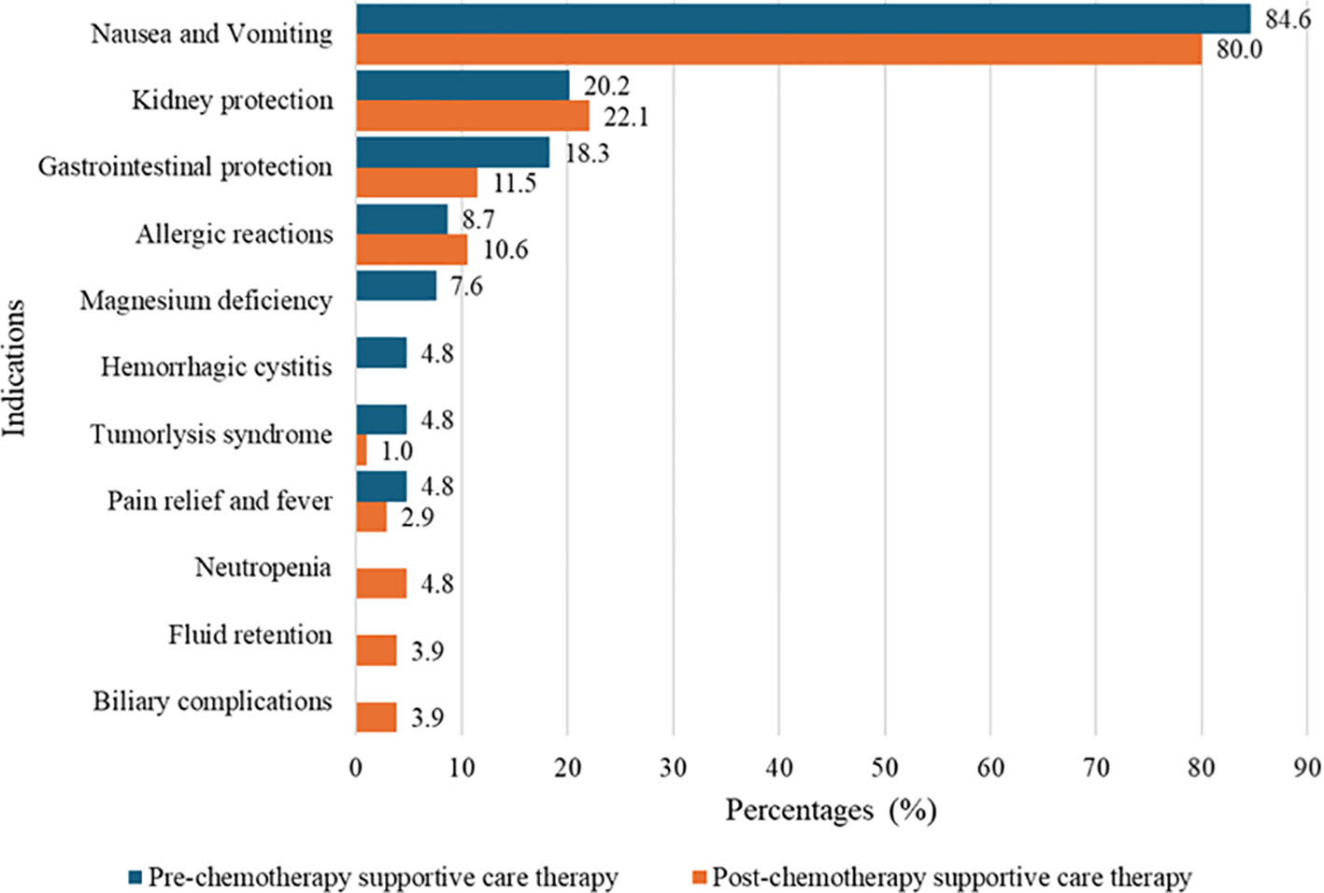
Indications of prescribed pre- and post-chemotherapy supportive care medications among pediatrics on chemotherapies at Bugando Medical Centre.

Various types of supportive care medications were prescribed to pediatric patients both before and after chemotherapies to prevent and manage chemotherapy-induced toxicities. The most commonly prescribed medication was the antiemetic serotonin receptor antagonist Ondansetron (5-HT3A), given as an IV injection prior to chemotherapy in 88 patients (84.6%) and as oral tablets post-chemotherapy in 84 patients (80.8%). Dexamethasone 3 mg/m², a corticosteroid often used as an antiemetic, was prescribed intravenously to 21 patients (20.2%) before chemotherapy and orally to 9 patients (8.7%) after chemotherapy. Hydration with normal saline was also commonly prescribed to protect against nephrotoxicity. Other supportive care medications, including pantoprazole, diphenhydramine, promethazine, paracetamol, and others, were prescribed based on the anticipated side effects of the chemotherapy regimens as shown in **Table 3**, while their specific indications during pre- and post-chemotherapy periods were as shown in **Figure 1**.

**Table 3:** Type and dose of supportive care medications prescribed Pre- and Post-Chemotherapy to children on chemotherapy at Bugando Medical Centre.

Type and Dose of Supportive Care Medication	Prescribed as Pre-chemotherapy supportive medication		Prescribed post-chemotherapy supportive medication	
	Formulation	Frequency (%)	Formulation	Frequency (%)
Ondansetron 0.45mg/kg	IV	88 (84.6%)	Oral	84 (80.8%)
Dexamethasone 3mg/m²	IV	21 (20.2%)	Oral	9 (8.7%)
Normal saline 125ml/m²	IV	21 (20.2%)	IV	19 (25.7%)
Pantoprazole 1mg/kg	IV	18 (17.3%)	–	–
Magnesium oxide 400mg	Oral	7 (6.7%)	–	–
Diphenhydramine 25mg	Oral	6 (5.8%)	Oral/IV	6 (5.8%)
Paracetamol 15mg/kg	IV	5 (4.8%)	Oral	2 (1.9%)
Mesna 600mg/m²	IV	5 (4.8%)	–	–
Allopurinol 10mg/kg	Oral	5 (4.8%)	Oral	1 (1.0%)
Promethazine 0.25mg/kg	IV/oral	4 (3.8%)	IV	1 (1.0%)
Metoclopramide 1mg/kg	IV	1 (1.0%)	Oral	1 (1.0%)
Famotidine 0.5mg/kg	Oral	1 (1.0%)	–	–
Hydrocortisone 1mg/kg	IV	1 (1.0%)	–	–
Diphenhydramine 25mg	IV	1 (1.0%)	Oral/IV	6 (5.8%)
S-Omeprazole/Omeprazole 20mg	–	–	Oral	12 (11.5%)
Neupogen 5mcg/kg	–	–	SC	5 (4.8%)
Ursodiol 10mg/kg	–	–	Oral	4 (3.9%)
Prednisolone 1mg/kg	–	–	Oral	4 (3.9%)
Furosemide 0.5mg/kg	–	–	IV	4 (3.9%)
Morphine syrup 0.5mg/kg	–	–	Oral	1 (1.0%_

IV, Intravenous administration; SC, Subcutaneous administration; Oral, Administered by mouth, -: Medication not prescribed during the respective period.

### 3.4 The pattern of supportive care medication prescribed in relation to the chemotherapy regimen prescribed

The majority of prescribed chemotherapy regimens 91 (87.5%) consisted of combinations of multiple chemotherapeutic agents, while a smaller proportion 13 (12.5%) were single-agent regimens. Additionally, most chemotherapy regimens were accompanied by supportive care medications to manage or prevent associated toxicities. The most commonly prescribed regimen was ABVD (Doxorubicin, Bleomycin, Vincristine, and Dacarbazine) to 11 (10.6%) patients, followed by CMV/COM (Cyclophosphamide, Methotrexate and Vincristine) to 10 (9.6%) patients. The supportive care prescribed with these regimens primarily included antiemetics (Ondansetron, Dexamethasone), hydration fluids (normal saline), and others for specific toxicities. Cisplatin-based regimens were associated with the greatest number of supportive care medications, with patients receiving up to eight supportive drugs, including magnesium oxide for managing magnesium deficiencies. Ifosfamide-based regimens were prescribed along with mesna and hydration to prevent hemorrhagic cystitis as shown in **Table 4**.

**Table 4:** Pattern of supportive care medication prescribed in relation to the chemotherapy regimen prescribed.

Chemotherapy regimens	Frequency	Percentage	Supportive care medication
Doxorubicin/Bleomycin/Vinblastine/Dacarbazine	11	10.6	Ondansetron, normal saline, dexamethasone, pantoprazole, paracetamol, omeprazole, allopurinol
Vincristine/Actinomycin D	10	9.6	Ondansetron, normal saline
Vincristine/Carboplatin/Etoposide	10	9.6	Ondansetron, normal saline, dexamethasone, pantoprazole, magnesium oxide, diphenhydramine, promethazine, neupogen
Vincristine/Methotrexate/Cyclophosphamide	10	9.6	Ondansetron, normal saline, dexamethasone, pantoprazole, prednisolone, s-omeprazole, allopurinol
Doxorubicin/Cyclophosphamide	7	6.7	Ondansetron, normal saline, dexamethasone, pantoprazole, magnesium oxide, diphenhydramine, promethazine, neupogen, ursodiol
Vincristine/6-Mercaptopurine/Methotrexate	7	6.7	Ondansetron, dexamethasone, s-omeprazole, omeprazole
Carboplatin/Etoposide	5	4.8	Ondansetron, dexamethasone, pantoprazole, diphenhydramine, promethazine, neupogen
Vincristine/Actinomycin D/Cyclophosphamide	4	3.9	Ondansetron, normal saline
Vincristine/Methotrexate	4	3.9	Ondansetron, normal saline, ursodiol
Carboplatin	3	2.9	Ondansetron, normal saline, dexamethasone
Doxorubicin/Cisplatin	3	2.9	Ondansetron, dexamethasone, pantoprazole, magnesium oxide, diphenhydramine, promethazine, furosemide, ursodiol, neupogen
Doxorubicin/Ifosfamide	3	2.9	Ondansetron, normal saline, dexamethasone, pantoprazole, mesna, furosemide
Etoposide/Cyclophosphamide	3	2.9	Ondansetron, paracetamol, morphine syrup, Neupogen
Rituximab	3	2.9	Ondansetron, normal saline, dexamethasone, pantoprazole, hydrocortisone, paracetamol, diphenhydramine,
Vincristine	3	2.9	Dexamethasone, allopurinol, prednisolone, s-omeprazole
Cisplatin	2	1.9	Ondansetron, normal saline, dexamethasone, pantoprazole, magnesium oxide, diphenhydramine, promethazine, metoclopramide, ursodiol
Vincristine/Actinomycin D/Cyclophosphamide	2	1.9	Ondansetron
Vincristine/Carboplatin	2	1.9	Ondansetron
Vincristine/Doxorubicin/Cyclophosphamide	2	1.9	Ondansetron, normal saline
Actinomycin D/Cyclophosphamide	1	1.0	Ondansetron, normal saline
Cyclophosphamide/Etoposide/Doxorubicin	1	1.0	Ondansetron
Cyclophosphamide	1	1.0	Ondansetron
Cytarabine/Daunorubicin	1	1.0	Ondansetron
Cytarabine/Etoposide	1	1.0	Allopurinol
Cytarabine/Etoposide/Ifosfamide	1	1.0	Ondansetron, normal saline, mesna
Cytarabine	1	1.0	Ondansetron, normal saline
Etoposide/Ifosfamide	1	1.0	Ondansetron, pantoprazole, mesna, prednisolone, furosemide
L-asparaginase	1	1.0	Paracetamol, diphenhydramine, famotidine
Vincristine/Cyclophosphamide	1	1.0	Ondansetron, prednisolone, s-omeprazole

### 3.5 Recommendations from guidelines and selected protocols used at Bugando Medical Centre

The cancer treatment guidelines and protocols used in making treatment decisions at BMC were selected based on the availability of information about their sources and years of publications. They were thoroughly read, and important information related to the current study was extracted. In the current National Cancer Treatment Guidelines (first edition, published in January 2020), eleven [11] types of childhood cancers are presented and their management elaborated. These are: brain tumors in children; leukemia in children; lymphoma in children; neuroblastoma; Wilm’s tumor; rhabdomyosarcoma; malignant bone tumors in children; retinoblastoma; germ cell tumor; hepatoblastoma; and nasopharyngeal carcinoma. It was observed that cancer presentation, pathophysiology, and treatment modalities (mention of regimens, types, and doses of chemotherapeutic agents) were all well described in a document, but information on chemotherapy-induced toxicities and their management was missing. Further assessment showed that the document has a separate section titled “Support Care for Cancer Patients” divided into two subsections: nutrition in cancer and oncological emergencies. In the nutrition section, the guidelines outline the impact of the disease and treatment on the nutrition status of a patient, as well as the influence of the patient’s nutrition status on tolerance of chemotherapeutic agents. The guideline does not mention or give any detail on the pharmacological management of nutrition deficiencies except for the use of iron supplements for anemia. Nevertheless, for oncological emergencies, the guideline defines the term as “any acute, potentially morbid, or life-threatening event directly or indirectly related to a patient’s tumor or its treatment.” It further suggests that “for prevention and early detection of oncologic emergencies, physicians must maintain a high degree of suspicion and adequately educate patients about preventive measures and the reporting of symptoms”, but the guideline does not mention chemotherapy-induced reactions or their management as oncological emergencies. The only pharmacological treatment mentioned is the management of symptoms of cord compression using dexamethasone or prednisolone, without elaboration on the dose and dosages. In contrast, two disease-specific guidelines, National Burkitt’s Lymphoma Treatment Guideline, published in 2015, and Clinical Guidelines for The Management of Children with Retinoblastoma, published in March 2016; had complete and lengthy descriptions of the management of CITs using supportive care medications. Generally, all the supportive care medications prescribed to patients with Burkitt’s lymphoma and retinoblastoma were according to these two guidelines.

On the other hand, out of over 30 different disease-specific protocols used at our cancer center, eight met the inclusion criteria and were selected. Upon thorough assessments, it was found that most of them contain little information with regard to supportive care medications but suggested prevention of CITs and monitoring of the patients for early detection and treatment of CITs as shown in **Table 5**.

**Table 5:** Descriptions of the supportive care medications in the disease-specific protocols at Bugando Medical Centre.

Name of the protocol	Type of cancer and (chemotherapies)	source	Edition year	Description of the supportive care medications
Modified UK2003/AALL0331	Standard risk ALL for interim maintenance I (8 weeks) (Vincristine, IV Methotrexate, IT Methotrexate)	BMC	8/8/2014	Suggests treating chemotherapy-specific ADRs by hydration and responding to specific symptoms, monitoring laboratory values, delay treatment in case of low blood corpuscles
Modified UK2003/AALL0331	Standard risk ALL for Delayed Intensifications (8 weeks) (Vincristine, Oral Dexamethasone, Doxorubicin, Cyclophosphamide, Thioguanine/6MP, Cytarabine, IT Metho)	BMC	8/8/2014	Suggests Monitoring blood corpuscles specifically ANC, not halting chemotherapy based on the presence of myelosuppression alone except when there is a serious infection. Emphasize pre-chemo infusion to reduce urine Specific Gravity from >1.015. Also, maintain hydration and use of furosemide once urine output is <3ml/kg/hr
AML-Protocol	Acute Myeloid leukemia (Excluding Acute Promyelocytic Leukemia) (IV cytarabine, IT triple- Meth, Cytarabine, Hydrocortisone)	Mulago Hospital	Not indicated	Suggests prevention of tumor lysis by hydration and use of allopurinol, the doses are clearly stipulated. Also suggests the use of antiemetics specifically ondansetron and metoclopramide as the first line but no description of dose and dosages. In addition, suggests the use of Co-trimoxazole as a prophylaxis for Pneumocystis carinii pneumonia (PCP) by taking BD doses on Saturday and Sundays until six months after the end of therapy
Protocol for Anaplastic large cell lymphoma	Non-Hodgkins lymphoma-anaplastic (Dexa, Cyclophosphamide, Triple IT)	Red cross children’s hospital	2003	Suggests hydration by 3L/m^2^/day Normal saline or dextrose saline with alkalization (although no alkalizer mentioned) and use of allopurinol
Protocol for treatment of children and adolescents with Hodgkin’s disease	Hodgkin’s lymphoma (Doxorubicin, Bleomycin, Vinblastine, Dacarbazine)	Muhimbili national Hospital	Not indicated	Suggests recognizing cardiotoxicity and fertility issues as a result of using chemotherapy. On these, it suggests considering the use of an optimum dose of anthracyclines because cardiotoxicity is a dose-dependent effect. The fertility issue is a concern in adolescence. The protocol suggests its awareness and consideration during treatment
Kaposi Sarcoma protocol	Kaposi sarcoma (Vincristine, Bleomycin, Doxorubicin)	Texas children’s hospital, Baylor International Pediatric AIDS Initiative	2010	Suggest that all patients should receive PCP prophylaxis for six months after completion of chemotherapy (although, no mention of medication for prophylaxis). It further suggests the use of stool softener specifically senna, bisacodyl, and docusate (written in brand name, colase) throughout the course of chemotherapy to avoid severe constipation and ileus secondary to vincristine

## 4 Discussion

The use of chemotherapies is a cornerstone treatment for over 95% of childhood cancers, either as the primary modality or in combination with surgery and/or radiation. While effective, chemotherapies often cause adverse effects that can limit their usefulness and deter patients from continuing treatment. Over 60 years of clinical experience and trials, scientists have identified these effects, leading to the use of supportive care medications to manage them (12). However, rational prescribing of supportive care medications is critical to minimizing chemotherapy-induced toxicities and improving patient outcomes (13).

In the current study, we analyzed 104 prescriptions of pediatrics at our center, where 150-250 children per year are actively undergoing cancer treatment according to institutional records. The majority of patients were young children aged between 3 and 6 years, Males and, diagnosed with nephroblastoma. This aligns with findings from other studies done on a similar group of patients at other cancer treatment centers in Tanzania. For instance, A study by Efraim et al. at Muhimbili National Hospital on the drug utilization pattern and adverse drug reactions of chemotherapy in pediatric patients identified nephroblastoma, B-cell acute lymphoblastic leukemia, Burkitt lymphoma, and retinoblastoma as the most common pediatric cancers (14). Additionally, our results are consistent with the Tanzanian Pediatric Cancer Network’s report titled “A comprehensive evaluation of the incidence of presenting patients and access to pediatric cancer care in Tanzania” which found nephroblastoma to be the most prevalent cancer among children undergoing treatment in Tanzania (15).

The predominance of stage II cancer, 36.5% (n = 38) in our study may reflect improved awareness campaigns in the region, leading to earlier referrals and diagnosis. This contrasts with findings from other Tanzanian centers, where most patients present with advanced-stage disease. For example, a study by Ester Majaliwa et al. at a tertiary hospital in northern Tanzania showed that over 75% of patients with childhood and adolescent cancers were diagnosed with cancer at stages III and IV (16). Similar trends have been reported in India, where the delayed diagnosis is often attributed to a lack of comprehensive clinical assessments and a focus on infectious disease as the primary cause of childhood illnesses as reported in a study by Mathew et al. (17).

Our study primarily examined the prescribing patterns of supportive care medications, which were categorized into pre- and post-chemotherapy use. Pre-chemotherapy medications, aimed to prevent acute adverse reactions, with ondansetron, 84.6% (n=88) and dexamethasone, 20.2% (n=21) being the most commonly prescribed to prevent nausea and vomiting. This observed dominance may be because ondansetron, a 5-HT_3_ receptor antagonist, is highly effective in preventing chemotherapy-induced nausea and vomiting (CINV), while dexamethasone enhances antiemetic efficacy, particularly in highly emetogenic chemotherapy regimens. These findings align with a review by Ganguly et al. on the pharmacology and optimization of therapy for nausea and vomiting (18). Normal saline infusions, 20.2% (n = 21) were used to protect kidney function, while pantoprazole 17.3% (n=18) was prescribed to prevent gastrointestinal ulcerations. Patients at risk of allergic reactions received diphenhydramine or promethazine (also has antiemetic properties), and those on cisplatin were given magnesium oxide to prevent hypomagnesemia. The use of these agents and other medicines relevant to specific toxicities in pediatric oncology patients is a common practice. The Children’s Oncology Group (COG) has endorsed clinical practice guidelines that recommend hydration protocols, including normal saline, to mitigate nephrotoxicity associated with certain chemotherapeutic agents. Additionally, proton pump inhibitors (PPIs) are often utilized to prevent gastrointestinal complications during chemotherapy. These practices are widely adopted to enhance patient safety and treatment efficacy (19). Patients in danger of developing tumor lysis syndrome were prescribed allopurinol to prevent uricemia and famotidine to prevent gastric ulcerative reactions from the effects of treatment. In addition, all patients on ifosfamide and some on cyclophosphamide were put on mesna. There is scant information from other centers in Tanzania about the best practices for managing and preventing chemotherapy-induced ADRs.

Post-chemotherapy, patients were primarily prescribed oral formulations of ondansetron (80.8%, n = 84) and S-omeprazole 8.7%, (n = 9) for delayed nausea and gastrointestinal protection, respectively. Hydration with normal saline 22.2%, (n = 23) was continued to mitigate nephrotoxicity, particularly in patients on platinum-based regimens. These practices align with findings from Ramalakshi et al., who reported similar supportive care strategies in adult cancer patients (20).

Our study highlights the need for standardized guidelines, as current practices are based on scattered evidence from various protocols. This could have been the reason for the irrational prescription observed, for example, despite the majority, 97% (n=101) of patients many of whom were on multi-agent being prescribed antiemetics, some patients 1.9% (n=2) on moderate-emetic-risk regimens (cytarabine, etoposide, and L-asparaginase) did not receive antiemetics, potentially leading to poor outcomes. This shows the importance of adhering to standard guidelines such as those from the American Society of Clinical Oncology (ASCO) and the National Comprehensive Cancer Network (NCCN) which recommend using at least three antiemetic classes for high-emetic risk chemotherapy (21). Additionally, the high number of supportive care medications prescribed to patients on cisplatin-based regimens (up to 8 medications) raises concerns about adherence and potential drug-drug interactions. Studies have emphasized the need to balance therapeutic benefits with the risk of polypharmacy in pediatric cancer care (22). A study by Eman Biltaj et al. on the supportive care medications associated with chemotherapy among children with hematological malignancy showed that there are potential pharmacokinetic conflicts that can arise between supportive care medications and chemotherapy. The study emphasized the importance of considering these interactions to optimize treatment outcomes for children. Therefore, there must be a balance maintained between what is likely to be achieved therapeutically and the prescription of several supportive medications (23).

We also observed, the medications such as diuretic furosemide 0.5 mg/kg, neupogen 5mcg/kg SC, prednisolone 1mg/kg PO, paracetamol 15mg/kg PO, and morphine, which were prescribed based on the presented signs and symptoms of fluid retention, anemia, and pain, respectively. This indicates that not only supportive medications are indicated for preventive purposes but also treatment, necessitating the availability of these medicines at all times of care.

On the other hand, our study observed the consistent lack of the current preferred and highly effective forms of supportive medications, such as palonosetron, a serotonin (5HT3) receptor blocker, and aprepitant, a neurokinin action blocker. These agents are highly preferred as shown by studies by Mathew et al. and Naohisa Yoshida et al. who reported that the use of palonosetron and aprepitant as post-chemotherapy treatments for nausea, was highly preferred over the use of ondansetron. The reason for this observation in our setting could be the issue of unavailability and inaccessibility due to their high cost compared to ondansetron (16, 20).

On another note, our findings reveal the importance of evidence-based practices in managing chemotherapy-induced toxicities. For example, magnesium supplementation for cisplatin-induced hypomagnesemia and mesna for ifosfamide-induced hemorrhagic cystitis were consistently prescribed, aligning with international recommendations (24, 25). However, the lack of unified national guidelines for supportive care in pediatric oncology remains a significant gap. Standardized, evidence-rich protocols are essential to optimize outcomes for children undergoing chemotherapy.

### 4.1 Conclusion

The majority of patients were prescribed supportive care medication to prevent and manage the side effects of chemotherapy treatment based on the available treatment guidelines and disease-specific protocols. However, many of these documents contain little to no information on the best prescriptions of supportive care medications. There is a need to assess the outcomes of the management of chemotherapy-induced adverse reactions using the prescribed supportive care medications.

## Data Availability

The original contributions presented in the study are included in the article/supplementary material. Further inquiries can be directed to the corresponding author.
